# Ethanol and Methanol Can Improve Huperzine A Production from Endophytic *Colletotrichum gloeosporioides* ES026

**DOI:** 10.1371/journal.pone.0061777

**Published:** 2013-04-17

**Authors:** Xin-Mei Zhao, Zhang-Qian Wang, Shao-Hua Shu, Wen-Juan Wang, Hai-Jie Xu, Young-Joon Ahn, Mo Wang, Xuebo Hu

**Affiliations:** 1 Institute of Medicinal Plant, College of Plant Science and Technology, Huazhong Agricultural University, Wuhan, China; 2 College of Life Sciences, Xinyang Normal University, Henan, China; 3 WCU Biomodulation Major, Department of Agricultural Biotechnology, Seoul National University, Seoul, Republic of Korea; University of Nottingham, United Kingdom

## Abstract

Huperzine A (HupA) is a plant alkaloid that is of great interest as a therapeutic candidate for the treatment of Alzheimer's disease. However, the current production of HupA from plants in large quantity is unsustainable because the plant resource is scarce and the content of HupA in plants is extremely low. Surprisingly, this compound was recently found to be produced by various endophytic fungi, which are much more controllable than the plants due to simpler genetics and ease of manipulation. However, it might be due to the innate properties of endophytic symbiosis, that production of this chemical in large quantity from endophytes has not yet been put into practice. Endophytic *Colletotrichum gloeosporioides* ES026 was previously isolated from a HupA producing plant and the fungi also proved to produce HupA. In this study, various fermentation conditions were tried to optimize the production of HupA from *C. gloeosporioides* ES026. Optimization of these parameters resulted in a 25.58% increase in HupA yield. Potato extracts supplemented with glucose or sucrose but not maltose facilitated HupA producing from the fungi. A final concentration of 0.5–2% ethanol stimulated the growth of fungi while methanol with the same treatment slightly inhibited the growth. However, both methanol and ethanol greatly increased the HupA production with the highest yield of HupA (51.89% increment) coming from ethanol treatment. Further analysis showed that both ethanol and methanol were strong inducers of HupA production, while ethanol was partially used as a carbon source during fermentation. It was noticed that the color of that ethanol treated mycelia gradually became dark while methanol treated ones stayed grey during fermentation. The present study sheds light on the importance of optimizing the fermentation process, which, combined with effective inducers, maximizes production of chemicals of important economic interest from endophytic fungi.

## Introduction

**Figure 1:**
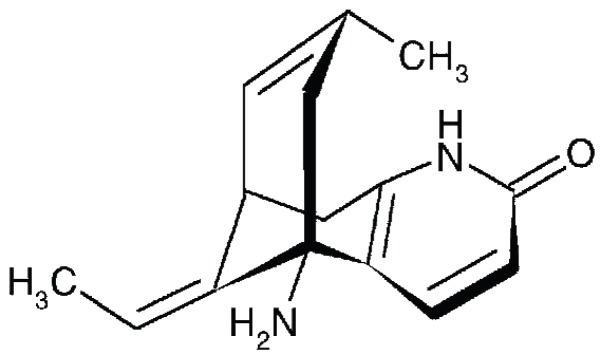
The chemical structure of Huperzine A.

HupA, a pharmaceutical Lycopodium alkaloid ((5R, 9R, 11E)-5-amino-11-ethylidene-5, 6, 9, 10-tetrahydro-7-methyl-5, 9-methano-cycloocteno[b] pyridine-2(1H)-one), was first isolated from the traditional Chinese medicine *Qian Ceng Ta* (the whole plant of *Huperzia serrata* (Thunb. ex Murray) Trev. (Lycopodiaceae)) by Chinese scientists (Figure 1) [1], [2]. HupA is an important compound that is used to treat Alzheimer's disease (AD) in China as a potent, highly specific and reversible inhibitor of acetylcholinesterase (AChE) with low toxicity [3]–[6].

*H. serrata*, the original source of HupA, actually possesses a very low content of HupA, i.e., 80 µg·g^−1^ cell dry weight (CDW) [7]. *H. serrata* belongs to a lower class of plants from Lycopodiopsida and it has a very limited distribution and an extremely long vegetative cycle. Naturally, it takes at least 15 years from spore germination through the gametophyte stage to finally reach the mature sporophyte stage [7]. Tissue culture for this plant is rarely successful (our own experience and correspondence with colleagues). Hence it is impractical to extract large quantity of the chemical from the plant for medicinal uses [7], [8]. In recent years, many more HupA-producing plants have been explored [7], [9]–[11]. While some other species in the Huperziaceae produce greater amounts of HupA, these species are even more rare in nature making them even less desirable candidates as natural sources for HupA production. So far, only two reports, by Szypulaa et al. [2005] and Ma and Gang [2008], have measured the HupA content of the cultivated plants [12], [13], but no successful commercial cultivation has been reported for *H. serrata* or other species in the Huperziaceae. On the other hand, although it is possible to chemically synthesize HupA [14], [15], the resulting racemic mixture has been found to be much less potent for AChE inhibition, compared to natural HupA derived from plant extracts [16]–[22]. The HupA on the pharmaceutical market is still predominantly extracted from plants, which results in the rapid decline of *H. serrata* in China due to over-harvesting [9].

Because of these trends, there is a keen interest in developing alternative methods to produce HupA. Since the taxol-producing endophytic fungi was successfully isolated from pacific yew in 1993, a large amount of compounds with new structures and various bioactivities were continuously discovered to be produced by endophytic microbe [23], [24]. Thus, to find a microbial source of HupA has attracted a wide spread of interest in recent years, and a few reports on the isolation of HupA-producing endophytic fungi from different Huperiaceae plants have been reported, such as *Acremonium* sp. 2F09P03B, *Blastomyces* sp., *Botrytis* sp., *Shiraia* sp. Slf14, *Cladosporium cladosporioides* LF70 and *Aspergillus flavus* LF40 [25]–[29]. In our study, a HupA-producing strain *C. gloeosporioides* ES026 was successfully isolated from a large variety of endophytic microbes of *H. serrata* (results to be published).

Although endophytic fungi offer a great opportunity to produce chemicals without the limitation of plant resources, especially for those rare and slow-growing plants, there are critical obstacles that need to be overcome for a meaningful microbial production. Stability of the microbial seeds is a basic requirement, however, the biggest challenge so far for the community. The progressive degeneration of strain vitality and the consistence of productivity of bioactive substances have long been troubles for many labs and so far, seldom have been reported to successfully produce active chemicals in large quantity from endophytic fungi. In this study, we looked into several factors that could possibly promote *C. gloeosporioides* ES026 to producing higher yield of HupA during the fermentation.

## Materials and Methods

### Materials

The methanol used for chemical purification was high-performance liquid chromatography (HPLC) grade level, and the rest of the chemicals were analytical reagent grade level. All of the chemicals and culture medium components were obtained from Sinopharm Chemical Reagent Co., Ltd., China. The HupA standard was supplied by Shanghai Tong Tian Biotechnology Co., Ltd. The acetylcholinesterase was supplied by Sigma-Aldrich.

### Fungal strain and culture conditions

The fungal *C.gloeosporioides* ES026, which produced the highest HupA in our initial screening, was preserved at the China Center for Type Culture Collection (CCTCC NO: 2011046), Wuhan, China. The strain was grown on potato-dextrose-agar (PDA) medium at 25°C for 7 days and the spores were collected from PDA slants with 0.05 M phosphate balanced saline (PBS) at pH 6.8. The spores were then washed, and diluted in sterile water and used as liquid seeds. For long term storage, the seeds were stored at −70°C in 17% glycerol. The liquid growth medium for the seeds consisted of (g/L of deionized water): 200 potato (soup extract), 5 peptone, 10 glucose or sucrose, 1.0 K_2_HPO_4_, 0.5 KCl, 1.0 MgSO_4_·7H_2_O, and was sterilized at 121°C for 15 min. To culture the seeds, every 100 ml culture medium was inoculated with 2×10^6^ spores ml^−1^ in a 500 ml flasks and cultured at 25°C with shaking at 200 revolutions per minute (rpm) for 48 hours. Prior to optimization and for the purpose of an initial determination of HupA activity, 50 ml of sterilized liquid medium in 250 ml Erlenmeyer flasks was inoculated with 1 ml of liquid seed culture. Each treatment of the culture was prepared in triplicate, and fermented for 7 days at 25°C with shaking at 150 rpm. For the fermentation study, the media was prepared and inoculated as described above for the seeds preparation, except the components were 200 g/L potato (soup extract), and 10 g/L sucrose with or without methanol or ethanol. The cultures were shaken at 150 rpm at 25°C for 3–8 days, depending on the purposes.

To recover the viability of the fungi previously cultured in a small volume for fermentation of seeds, the fungi were first grown in slants made of cold water extract from host plants for 7 days at 25°C. The fungi was then transferred into basic media (0.5% carbon sources plus 100 g/L potato soup extract) for 4 days and rich media (1.5% carbon sources plus 200 g/L potato soup extract) for an additional 3 days. The slants were then kept in 4°C and used as sources of seeds for regular fermentations.

### Optimization of growth parameters in submerged fermentation

The influence of fermentation temperature on HupA production was investigated by incubating the culture at temperatures ranging from 15 to 30°C, each at 5°C increment. The effect of the fermentation period at each individual temperature was also assessed by analyzing the HupA yield at various time points. These optimized parameters were then used to investigate the effect of different inducers on HupA production. The biomass production was measured as CDW per liter of culture broth. The yield of HupA from different treatments was determined by HPLC as follows.

### HupA induction by methanol and ethanol

Filter sterilized methanol and ethanol were added into the potato-dextrose liquid as elicitor respectively, to a final volume ratio of 0.5%, 1.0% 2.0%, and 3.0% prior to the inoculation. Subsequently, shake flask fermentation was carried out according to the optimized growth parameters.

### Preparation of fungal extracts

The fermented mycelia were harvested by centrifugation at 12 000 rpm for 10 min after being rinsed 3 times with distilled water. The mycelia was then dried out at 40°C overnight and grounded into powder. To extract the chemical, each sample of raw material (1.0 g) was extracted with 0.5% hydrochloric acid (*w/v*, 30 ml) overnight, followed by ultrasonication in a water bath at 40°C for an hour. Next, the extracts were filtered and the filtrates were rendered with ammonia solution to pH 9.0. About one hour later the water phase was extracted three times with CHCl_3_, and then the combined CHCl_3_ extracts were evaporated to dryness under reduced pressure. The dry residue was then dissolved in 1.0 ml methanol and passed through a 0.45 µm polytetrafluoroethylene syringe filter into a 2.5 ml measuring vial prior to RP-HPLC analysis. Statistical analysis was performed using Microsoft Office Excel 2003.

### HupA quantification by high performance liquid chromatography (HPLC)

The measurement of HupA content was performed by HPLC using a DIONEX UltiMate 3000 system (Thermo Scientific, U.S.) and the data were processed and analyzed with Chromeleon Analysis Software (revision 6.8, Thermo Scientific, U.S.). Chromatographic separations were achieved on Acclaim® C_18_ columns (4.6 mm×250 mm, 5 µm Thermo Scientific, U.S.). The temperature of the column compartment was kept at 40°C. The injection volume was set to 20 µl. The flow rate was 1.0 ml/min using 80 mM ammonium acetate (pH 6.0)-methanol (7∶3, *v/v*) as mobile phase. The effluent was monitored at 310 nm. Quantification was achieved by using the standard curve generated from the HupA standard in a concentration range of (0.5–8.0) mg·L^−1^, where the peak area and height showed linear correlation with the absorbance (R^2^ = 0.9995).

### Detection of HupA activity

The HupA purified by High-speed Countercurrent Chromatography (HSCCC) (Shanghai Tontian Biotech., China) was used to detect AChE inhibition activity *in vitro* based on the previous methods with some improvement [30]. Briefly, a pre-incubation volume of 100 µl 0.1 M saline buffer (pH 7.2) containing test sample plus reference standard of various concentrations, 10 µl of the substrate (0.1 M acetylthiocholine iodide in 10 ml of phosphate buffer) and 10 µl of enzyme (1 U ml^−1^). In the control, the test sample was replaced by equal volume of methanol and the rest was kept the same as above. The mixture was incubated for 15 min at 25°C. Next, 100 µl of 10 mM (5, 5′-Dithio bis-(2-nitrobenzoic acid), DTNB), (3.96 mg of DTNB dissolved in 10 ml phosphate buffer pH 7.2) was added to the mixture and incubated for another 5 min at 25°C. The color development was then measured in a micro-well plate reader at 412 nm (Bio-Rad, Hercules, CA, USA). The percentage of inhibition was calculated using the formula: (control absorbance-sample absorbance)/control absorbance×100%.

### The counting of conidiophores

The conidiophores were separated from mycelia and counted in a Neubauer hemacytometer. When the cells reached a certain time point of culture, the conidiophores in the media were separated by a layer of fabric cloth of 100 meshes on top of a piece of Grade 3 Whatman filter paper using a vacuum assisted Buchner funnel. The flow-through was then spun down at 10,000 rpm for 5 min. The pellet was resuspended in PBS (pH 6.8) at one-tenth of the starting volume. Different dilutions were made and the number of conidiophores was then counted using a hemacytometer. Each sample was repeated three times.

## Results

### Maintenance of the stability and viability of the HupA producing endophytic fungi

A variety of endophytic fungi was isolated and cultured, and the HupA yield was then measured, irrespectively. One HupA-producing strain was identified as *C. gloeosporioides* ES026, which produces the chemical approximate 25.47 µg·g^−1^CDW of mycelia when the fungi were cultured in 28°C for 6 days. We designate this value as the initial level of HupA and thereafter various means were tried to improve the productivity in this study. The fungi conidiophores were kept in −70°C for long term storage as seed resources for later research. To monitor the viability and stability of fungi, a total of 9 continuous passages of liquid and then solid culture were conducted and the HupA production was measured. The mycelia yield and HupA production started to show obvious decline in the 7^th^ passage. In the 9^th^ passage, the mycelia turned from white to yellow after 48 hours of culture and both of the mycelia yield and HupA content were significantly lower than the initial value (data not shown). To overcome the problem, the cells were first grown in host plant extract and then in basic and rich media. This strategy helped the maintenance of both the viability and stability of the fungi. We have been able to keep the viability and stability of the fungi without limitation for a period of 3 years, which is our experimental span thus far. Since there is no traceable difference between fungi stock made in early and late passages (up to the sixth passage), we regularly used the stock made by recovery preparation for the rest of fermentation study.

### Effect of fermentation conditions on the HupA production

**Figure 2:**
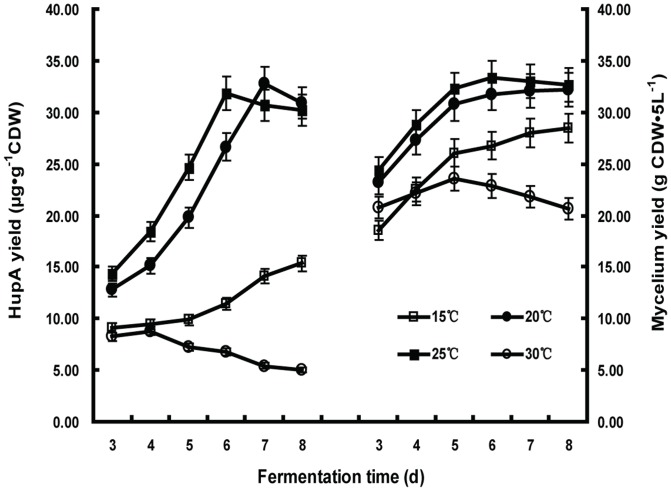
The effect of fermentation duration on HupA yield and biomass at various temperatures. *Colletotrichum gloeosporioides* ES026 was cultured with the liquid growth medium consisting of potato 200 g/L (soup extract) and sucrose 10 g/L. Each data series represents a fermentation carried out at a specific temperature, with open circles (○) representing 30°C, closed squares (▪) 25°C, closed circles (•) 20°C, and open squares (□) 15°C. The cells were shaken at 150 rpm for the indicated culture time. The biomass and HupA yield were then quantified. Results are the mean of triplicates with standard deviation represented by error bars.

The HupA yield from different growth temperatures and incubation times was first investigated. The results presented in Figure 2 indicated that HupA production was simultaneously affected by incubation temperature and duration. *C. gloeosporioides* ES026 produced the most HupA at elevated temperature (32.75 µg·g^−1^CDW at 25°C) while it produced the least at 15°C (15.35 µg·g^−1^CDW). The results also showed that at higher fermentation temperatures, a shorter fermentation time is required for optimal HupA production (6 days at 25°C versus 7 days at 20°C). However, when the fermentation temperature increased to 30°C, HupA production was significantly lower than that under lower temperatures and appeared to decline with the extension of fermentation time. The HupA yield closely correlates with the mycelia production, where fermentation at 25°C for 6 days produced the highest mycelia amount, meaning a better nutritional growth promotes the chemical production. The fungi prefer a cooler growth temperature as it is the ambient temperature for the growth of the host plant. In our experiments, only the mycelia balls were collected for the measurement of HupA yield because dispersive mycelia were hard to collect from the thick culture. Only trace amounts of HupA is present in the remaining media (data not shown). We noticed that the shaking speed around 150 rpm helps to form better mycelia balls and thus to improve the HupA yield.

The pH for the culture of *C. gloeosporioides* ES026 was optimized by examining the effect of different initial culture pH values of 4.5, 5.0, 5.5, 6.0 and 6.5 in a parallel experiment. The results showed that the yield of fungi mycelia was highest when the pH was 6.0 and 6.5 (data not shown). In order to monitor the possible pH shift during fermentation, the pH values from a total of 19 batches of fermentation were recorded on the day 7 of culture. The pH range of all samples was between 3.95 and 5.33. Among them, 56% of the samples produced a higher HupA yield than the average from each batch, and the pH range for these samples was between 4.71 and 5.05. It is possible to adjust the pH during the culture but it increases the likelihood of contamination. We chose to use the potato soup extract based media without adjusting the pH, which was around 6.2 after autoclaving. Other parameters, such as the length of light per day and inoculation ratio, which may also affect the mycelia growth and the HupA yield, were not tested individually, are listed in Table 1.

**Table 1:** Summary of optimized cultivation parameters in shake-flask fermentation.

Optimized Parameters	*C. gloeosporioides* ES026
Fermentation temperature (°C)	25°C
Fermentation duration (d)	6
Shake-flask shaken at (rpm)	150
Volume of liquid (*v/v*)	1∶5
Inoculum volume (%, *v/v*)	2
Length of light per 24 hr (h)	9
Optimized HupA production (µg·g^−1^CDW)	32.75
Relative increase (%)	28.58

### Effect of carbon sources on HupA production

**Figure 3:**
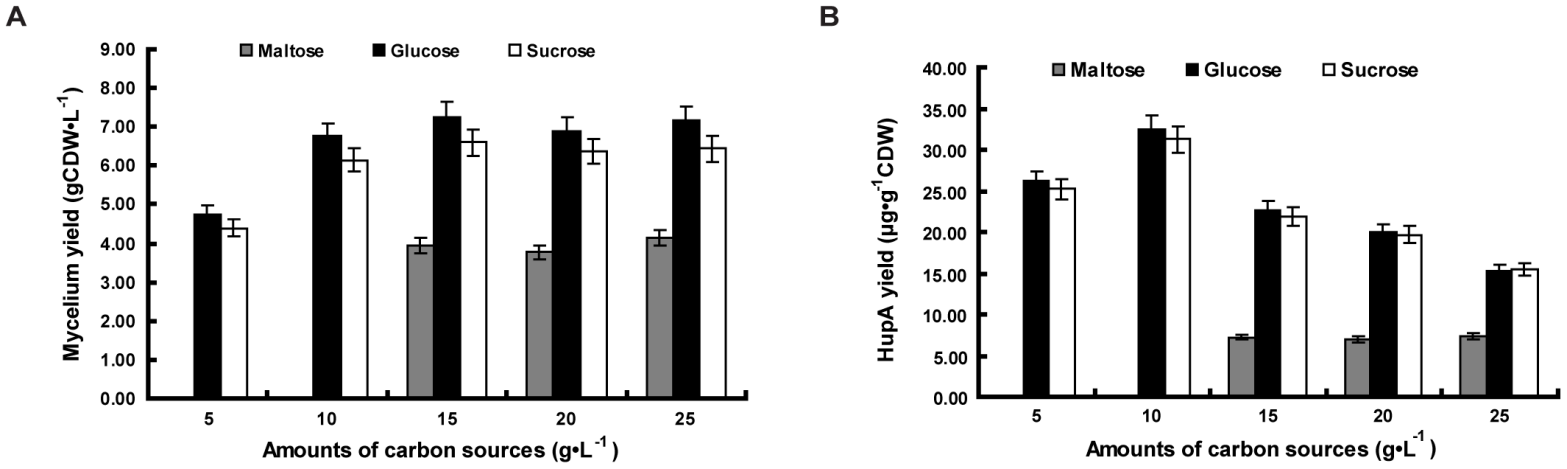
The effect of different carbon sources on biomass and HupA yield of *Colletotrichum gloeosporioides* ES026 cultures. The fungi were cultured in the liquid growth medium consisting of potato 200 g/L (soup extract) and maltose, glucose or sucrose, which were added at indicated amounts. The culture was shaken at 150 rpm at 25°C for 7 days. The biomass (A) and HupA yield (B) were then quantified. Each data point is the average of three replicates with *error bar* representing the standard deviation.

In this experiment, the influence of supplemental carbon sources and nitrogen sources to the primary substrate (potato extracts) on HupA production was assessed. Three different carbon sources, maltose, glucose and sucrose, each added in three different concentrations, were compared. Of the three, the addition of maltose significantly lowered HupA production and mycelia yield, while the addition of glucose and sucrose produced comparably higher amounts of both mycelia and HupA (Figure 3). It was also noticed that comparatively lower concentrations of glucose or sucrose produced higher amounts of HupA, while the mycelia production remained the same level (Figure 3). Different nitrogen sources, including ammonium nitrate, ammonium sulfate, yeast extract and beef extract, had no significant effect on HupA or mycelia production (data not shown).

**Figure 4:**
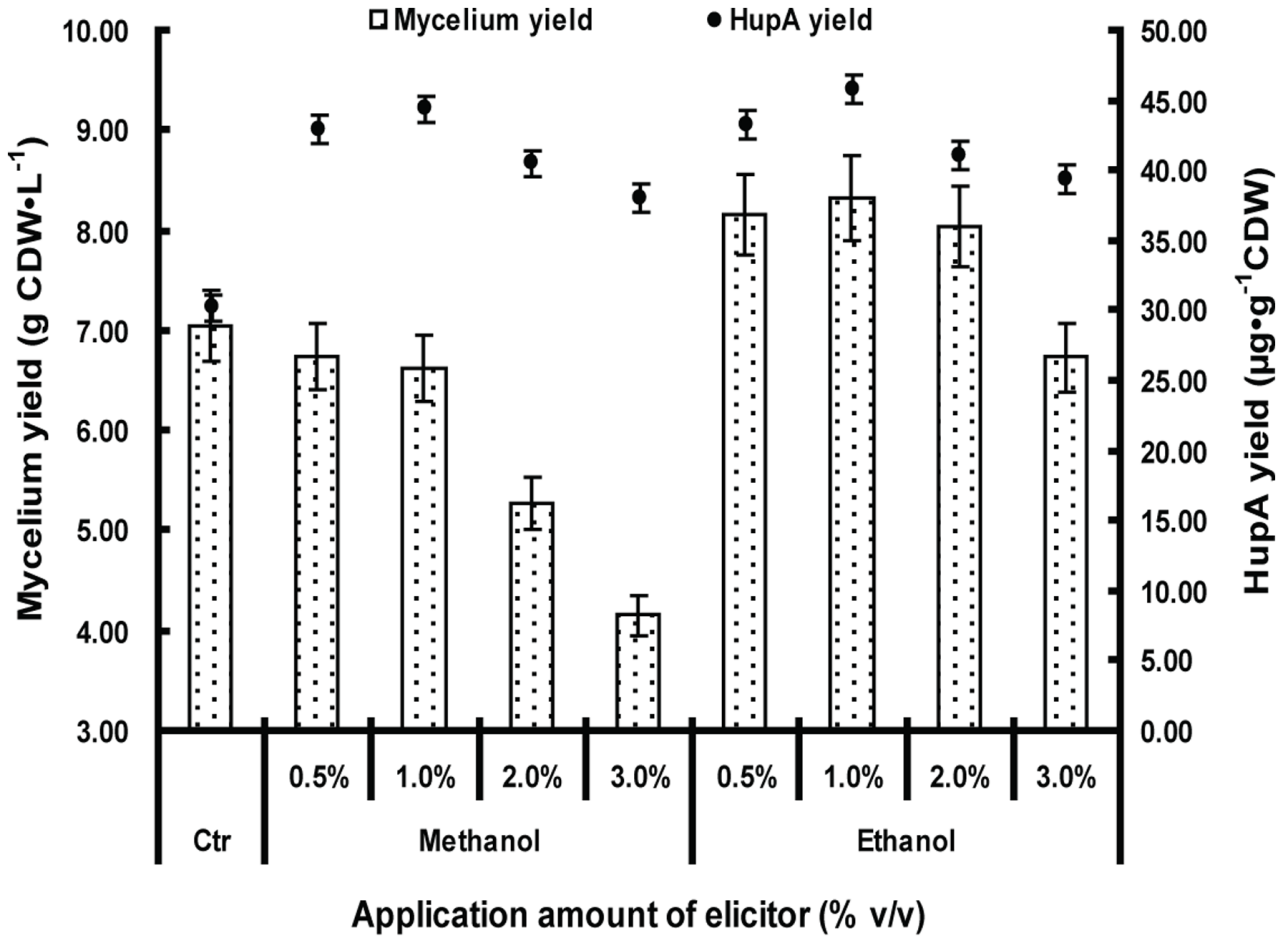
The effect of elicitors on HupA yield and biomass. *Colletotrichum gloeosporioides* ES026 was cultured in liquid growth medium consisting of potato 200 g/L (soup extract), sucrose 10 g/L and methanol or ethanol was added as indicated amounts, with a blank control (Ctr). The culture was kept shaking at 150 rpm at 25°C for 7 days. The biomass and HupA yield were then quantified. Results are the mean of triplicates with standard deviation represented by error bars. HupA production and biomass were assessed at fermentation day 7.

In some cases of microbial fermentation, methanol and ethanol are used as carbon sources for fungi and bacteria under adverse conditions [31]–[34]. We reasoned that *C. gloeosporioides* ES026, as an endophytic microbe which dwells inside of the plants, might be accustomed to oxygen deficiency. Because anaerobic respiration normally results in the alcohol fermentation [35], we decided to test whether methanol and ethanol affect HupA production. Methanol and ethanol were first added to potato-dextrose fluid medium at different concentrations. Compared to the contrast (30.16 µg·g^−1^CDW), HupA production from these two treatments significantly increased (Figure 4). The highest HupA production from ethanol treatment increased by 51.89% (45.81 µg·g^−1^CDW) when 1% ethanol was added. The HupA production went down when higher amounts of ethanol were added. The addition of methanol similarly influenced the HupA yield. The addition of 1% methanol also promoted HupA production with the highest amount increase to 47.05% (44.35 µg·g^−1^CDW) above the control level. Compared to the control (7.05 gCDW·L^−1^), mycelia production was significantly lower when methanol was added at all ratio, with the lowest to 41.13% of the control when 3.0% methanol was added (4.15 gCDW·L^−1^). Ethanol had a positive effect on mycelia growth when the application amount was between 0.5% and 2.0%, while it decreased growth by 4.54% when 3.0% was applied (6.73 gCDW·L^−1^). Due to these results (Figure 4), both ethanol and methanol were added to 1.0% in the subsequent experiments for the highest yield.

**Figure 5:**
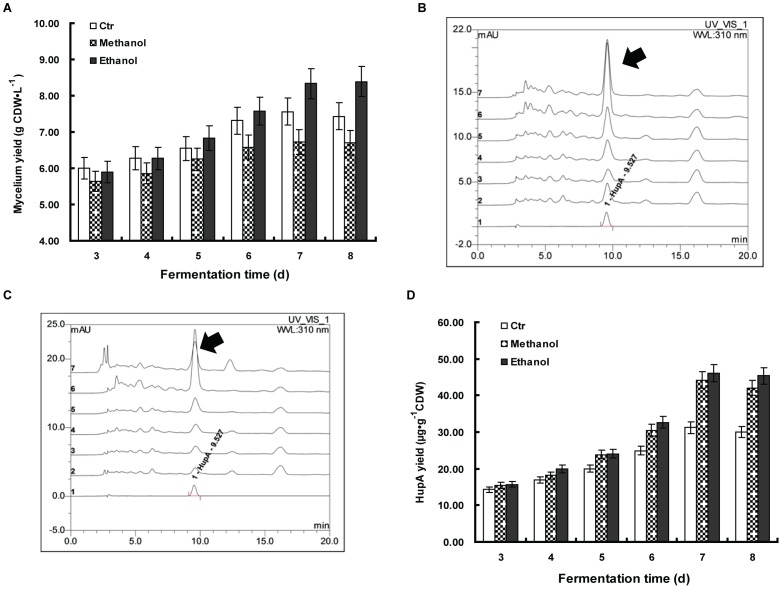
The effect of 1% methanol and 1% ethanol on *Colletotrichum gloeosporioides* ES026 fermentation in a time course study. *Colletotrichum gloeosporioides* ES026 was cultured in liquid growth medium consisting of potato 200 g/L (soup extract), sucrose 10 g/L and 1% methanol or 1% ethanol, with a blank control (Ctr). The culture was kept shaking at 150 rpm at 25°C for the indicated incubation time. The mycelium biomass was then compared among the treatment groups (A). The HupA yield with ethanol (B) and methanol (C) treatment was analyzed by HPLC of which, Curve 1 depicts authentic HupA (2.0 µg·ml^−1^), while curves 2 to 7 represent the HupA from 3 to 8 days of fermentation, respectively and the yield was quantified (D). The arrows indicate the peak for HupA. Each data point is the average of three replicates with *error bar* representing standard deviation.

In an independent experiment, we investigated the time course of the *C. gloeosporioides* ES026 fermentation when 1% methanol and ethanol were added, irrespectively. It showed that ethanol improved the mycelia formation since the mycelia yield increased steadily and overtook the control from day 3 to day 8, with the maximum at day 8. While methanol had no obvious effects on the mycelia yield, compared to the untreated control (Figure 5A). The extracted crude HupA from different fermentation times was then loaded into HPLC for quantification (Figure 5B, 5C). The HupA yield correlated with the mycelia growth, as both methanol and ethanol promoted the HupA production for up to 8 days of fermentation. The maximum increase of the yield for both methanol and ethanol was in the day 7, with a 47.55% (46.05 µg·g^−1^CDW) for ethanol and a 41.72% (44.23 µg·g^−1^CDW) for methanol, respectively (Figure 5).

**Figure 6:**
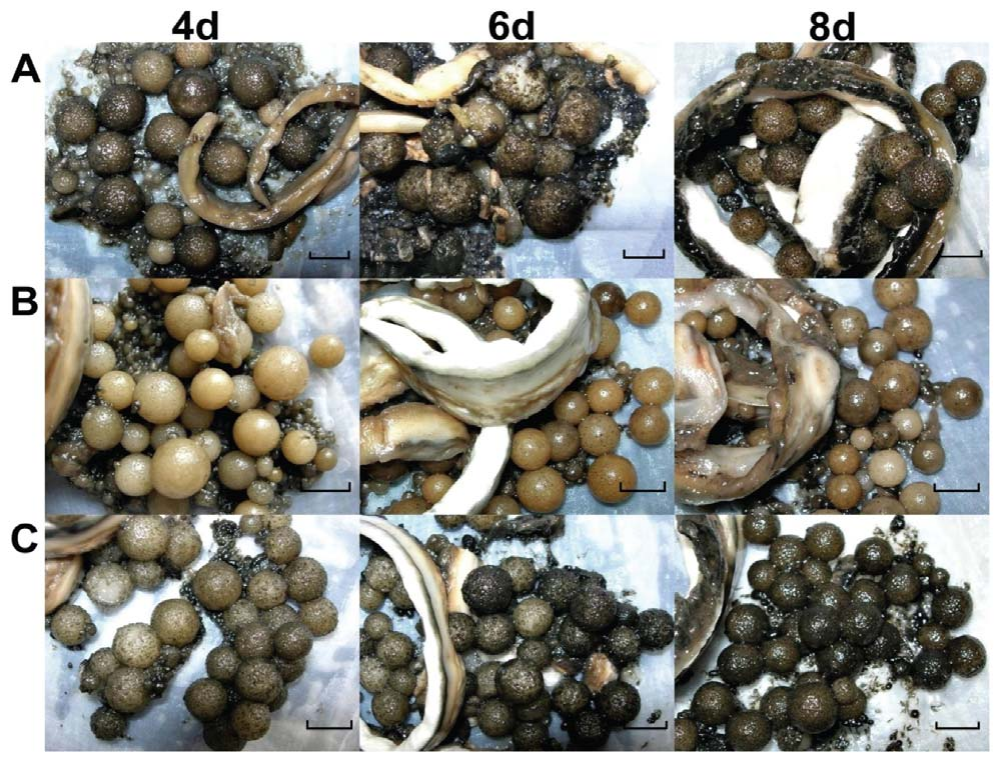
The morphometrics of mycelium pellets of *Colletotrichum gloeosporioides* ES026 from different fermentation time. The fungi were cultured in liquid growth medium consisting of potato 200 g/L (soup extract), and sucrose 10 g/L at 150 rpm shaking at 25°C. The treatment included the media itself (A) and 1% methanol (B) or 1% ethanol (C). On the days 4, 6 and 8, samples were harvested and the sizes were measured with a vernier caliper. (*bar* = 1.0 cm).

To investigate the difference between ethanol and methanol on the induction of HupA, the morphology of the mycelia was recorded at different times of induction. It showed that mycelia pellets were in two forms, the globular shape and the belt-like one. These two shapes were present when it was whether induced by ethanol, methanol or un-induced (Figure 6). The sizes of mycelia balls of three different treatments were very close at days 4, 6 and 8. The color was the only visible difference among these three treatments. The pellets in the methanol treatment displayed a golden color during the fermentation, while the non-induced control and the ethanol treated pellets were grey in color and became darker from day 4 to day 8 (Figure 6).

### Methanol and ethanol as sole carbon source

**Figure 7:**
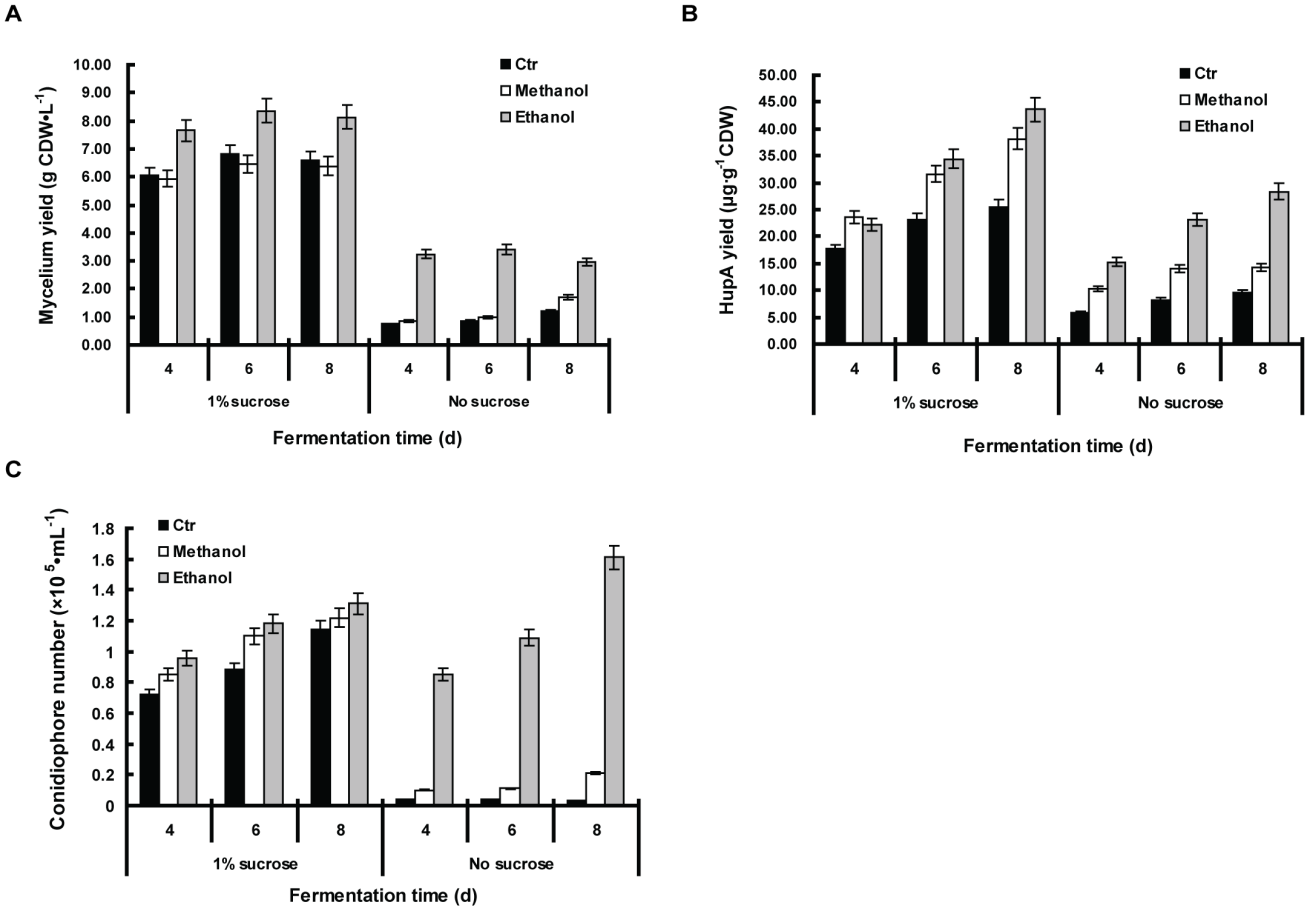
Effect of 1% methanol and 1% ethanol on *Colletotrichum gloeosporioides* ES026 fermentation with or without sucrose in the media. The fungi were cultured in liquid growth medium consisting of potato 200 g/L (soup extract) with or without sucrose. To the culture was also added with 1% methanol, 1% ethanol or a blank control (Ctr). The culture was shaken at 150 rpm at 25°C for the indicated incubation time. The mycelium yield (A), HupA yield (B) and conidiophore amount (C) with different fermentation time were shown. The data set presents the result from fermentation days 4, 6, 8 only. Each data point is the average of three replicates with *error bar* representing the standard deviation.

To further elucidate the beneficial role of methanol and ethanol in the fermentation, another set of experiment was conducted with or without the sucrose as a main carbon source (Figure 7). As shown in Figure 7A, compared to 1% sucrose, mycelia yield was greatly reduced when sucrose was depleted in the media, and the HupA yield was altered accordingly (Figure 7B). We checked the conidiophore number in each of the culture media and it indicated that the numbers were significantly lower if no sucrose was added, except in the case when ethanol was present (Figure 7C).

### AChE inhibition activity assay of fungal HupA

In order to ascertain that the chemicals we harvested from the mycelia were the same as the HupA commercial standard, we run HPLC with the same amount of fraction of HupA purified from the mycelia, which was induced by ethanol or methanol. The standard was also run as a control. As it can be seen that, from day 3 to day 7, all of the purified samples contain a peak corresponding to the standard and the height of the peak closely correlates with the yield of HupA reported previously (Figure 5B, 5C). Therefore, we concluded that the HupA was harvested in the right time point.

**Figure 8:**
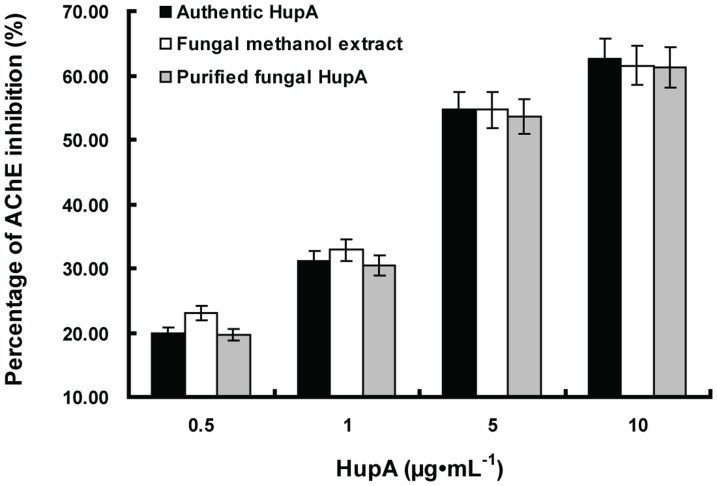
The inhibitory effects of authentic HupA, Fungal methanol extract and fungal HupA purified by HSCCC on acetylcholinesterase activity *in vitro*. Values are mean ± SEM expressed as percentage inhibition.

The AChE inhibition activity of HupA purified from ethanol induced fermentation and its methanol crude extract, was compared with authentic HupA. As HupA concentration increased from 0.5 to 10 µg·ml^−1^, the percentage of AChE inhibition increased. The inhibitory effect of purified fungal HupA by HSCCC against AChE activity was similar to authentic HupA. However, it is worth mentioning that the methanolic extract of strain *C. gloeosporioides* ES026 obviously exhibited stronger inhibitory activity than authentic HupA and the purified fungal HupA when the inhibitors concentration were 0.5 µg· ml^−1^(Figure 8).

## Discussion

Screening of endophytic fungi from pharmaceutical plants to produce drugs has been popular in the past ten years, and has led to the identification of dozens of endophytic microbes of various species [23], [24]. These microbes are ideal replacements for their corresponding plants to generate the drugs since they are simpler in genetics, easier to manipulation and more cost-effective. In spite of its promises, continued efforts to optimize the use of microbes to generate drugs have not been reported in recent years. Based on our own experience and correspondence with colleagues, there are still obstacles impeding the progress, which some are thought to be simple to overcome. It has also been reported that several endophytic HupA producing fungi have been isolated from the host [25]–[29], but no following work has been documented so far. Under this context, our present report detailed a stepwise approach to optimizing HupA production with various growth parameters of the strain *C. gloeosporioides* ES026, which was previously screened by our lab (results to be published). Special attention was given to the beneficial effect of ethanol and methanol when they were used as additives.

As mentioned in the introduction, a fundamental obstacle for the utilization of endophytic fungi is to maintain the vitality and stability of the cells during passages. In our hands, both the vitality and stability of *C. gloeosporioides* ES026 went down after seven passages. However, these properties remained when the cells younger than six passages were cultured with the host extract or re-inoculated and re-isolated to and from the host. It is logically reasonable if a certain extent of interdependence to be built among the endosymbionts. However, little is known about what is needed from the host for a consistent chemical production. We are now testing extracts from plants with different genetic distance to the original host, in the hope to find more sustainable alternatives or chemicals dominating the dependence. Nevertheless, the current strategy stabilized the HupA production with a small quantity of host plants.

In the process of analysis of HupA yield, we found that only a trace amount of the chemical is released into the media (data not shown), indicating that most of the HupA is confined to the inside of the cells. However, it is very difficult to collect the dispersed mycelia in the media when the culture reaches a high density. We noticed that many mycelia balls with a radius of approximately 1 cm will form if the shaking speed is set to about 150 rpm (Figure 6). The mycelia balls can be simply collected by filtration and they contained a substantial yield of HupA. Since the HupA is largely derived from the mycelia balls and the shaking speed is lower than in standard microbial culture, which is normally around 225 rpm, we posit that the lower speed is a balance of two key factors of the fermentation, the shear stress and dissolved oxygen tension. Higher shaking speed promotes oxygen distribution for microbial metabolites in general [36]–[39], but it also breaks the mycelia ball formation. Therefore, a lower speed favors the best mycelia growth and HupA yield.

The optimum growth temperature for the *C. gloeosporioides* ES026 is 25°C in culture. The mycelia yield is remarkably lower when it is cultured at 15°C or 30°C (Figure 2). Prolonged incubation beyond the optimal HupA production period resulted in a steady decrease HupA yield. Shorter incubation time is generally preferred when the overall cost of fermentation is considered in a large-scale process and it reduces the potential of contamination [40], [41].

*C. gloeosporioides* ES026 exhibits a striking difference on the utilization of carbon sources. *Saccharomyces cerevisiae*, the model species of yeast, can quickly shift to a maltose-addictive metabolic feedback when the glucose is not readily available [42]. However, *C. gloeosporioides* ES026 utilizes maltose far less efficiently than either glucose or sucrose (Figure 3). From an evolutionary point of view, endophytic symbioses are widely prevalent [43] and the symbioses might have formed a long time ago between *C. gloeosporioides* ES026 and its host, and thus it shaped a unique trait of maltose metabolism, which is largely different from the surface grown opportunistic fungi *S. cerevisiae*.

In this study, we showed that 1.0% ethanol but not methanol can promote mycelia production up to 18.01% and both of them promoted the HupA production. It has been reported that methanol and ethanol can be used as a carbon source for fungi but it is under the pressure of extreme stress, namely, either methanol or ethanol is consumed when no other prior carbon source such as glucose or sucrose is present. In our study, the methanol or ethanol was added along with glucose or sucrose, meaning that these two were possibly not consumed as a carbon source (Figure 4, 5). This could clearly be seen from the methanol treatment, of which 3% methanol produced half of the amount of mycelia than that of control, but on the contrary, its HupA yield is about 25.96% higher than that of the control (Figure 4). It indicates a possible connection of methanol or ethanol treatment to HupA metabolism. In our study, 1% methanol or ethanol exhibit highest HupA production, which correlates with many other studies in which the concentration of 1% of these two chemicals is most amicable for fungi growth [44], [45].

The contribution of methanol and ethanol to *C. gloeosporioides* ES026 growth and HupA production is clear, however, the mechanism by which the alcohols promote these outcomes remains uncertain. When no additional carbon source besides the PDA nutrients was present, the fungi hardly grew in control as well as in methanol added media (Figure 7), but did grow in the presence of ethanol, albeit to a lesser extent than in the presence of 1% sucrose (Figure 7). This clearly shows that methanol was not used as carbon source, while ethanol could be used as a less efficient main carbon source. Hence, both of ethanol and methanol contribute the HupA production largely by a function other than carbon sources. As many of research indicated that ethanol and methanol are a cellular stress for plants [46]–[47], it can be postulated that the function of HupA might be related to the endophytic symbiosis or the host acclimatization.

To our knowledge, this is the first report on the enhancement of HupA isolated from the culture mycelia of *C. gloeosporioides* ES026 by using ethanol or methanol as an inducer. The fungi might have formed a unique secondary metabolism, of which, methanol and ethanol acts as a positive regulator of the HupA production. It will be very intriguing to dissect the pathways involved in HupA synthesis and regulation. The expression sequence tag analysis of *H. serrate* screened more than a thousand unique transcripts, many of which were involved in the chemical secondary metabolites [48]. We are now sequencing the whole expression profile of the *C. gloeosporioides* ES026 and hope it will assist our understanding of the HupA biosynthesis and regulation, if compared to the unique host transcripts. In the near future, we also plan to examine the molecular cascades of HupA production upon methanol and ethanol induction.
